# Sarcopenia in Patients with Chronic Thromboembolic Pulmonary Hypertension

**DOI:** 10.3390/jcdd12050162

**Published:** 2025-04-22

**Authors:** Steven Hopkins, Jillian Hall, Hollie Saunders, Riyaz Bashir, Vladimir Lakhter, Anjali Vaidya, Ahmed Sadek, Paul Forfia, Estefania Oliveros

**Affiliations:** 1 Internal Medicine Department, University of Pittsburg Medical Center, Pittsburgh, PA 15219, USA; hopkinss6@upmc.edu; 2 Pulmonary Hypertension, RHF and CTEPH Program, Temple Heart and Vascular Institute, Philadelphia, PA 19140, USA; jillian.hall@tuhs.temple.edu (J.H.); hollie.saunders@tuhs.temple.edu (H.S.); riyaz.bashir@tuhs.temple.edu (R.B.); vladimir.lakhter@tuhs.temple.edu (V.L.); anjali.vaidya@tuhs.temple.edu (A.V.); ahmed.sadek@tuhs.temple.edu (A.S.); forfiap@gmail.com (P.F.); 3 Department of Cardiology, Columbia University Irving Medical Center, New York Presbyterian Hospital, New York, NY 10032, USA

**Keywords:** CTEPH, pulmonary hypertension, pulmonary thromboendarterectomy, sarcopenia, chronic thromboembolic pulmonary hypertension

## Abstract

Background: Sarcopenia, or loss of skeletal muscle mass, has been associated with poor outcomes (e.g., functional decline, increased mortality, and low quality of life), but its role in CTEPH remains unclear. The psoas muscle index (PMI) is a validated measure of sarcopenia. We investigated the incidence of sarcopenia using PMI in CTEPH. Methods: Retrospective analysis of a single-center cohort of patients with CTEPH with an available computed tomography of the abdomen and pelvis (CTAP). PMI was measured at the L3 level of the CTAP and was then calculated using the formula (left psoas area + right psoas area/height^2^). Patients in the first quartile of PMI were classified as sarcopenic. Results: We reviewed 558 patients with CTEPH, and 97 patients had an available CTAP before intervention. Sarcopenia was identified in 26 (24.8%) of the patients and was associated with worse baseline functional status (*p* = 0.008), higher mean pulmonary artery pressure (48 vs. 39 mmHg; *p* = 0.002), and higher pulmonary vascular resistance (9.9 vs. 6.8 WU; *p* = 0.013). Post-PTE, patients with sarcopenia exhibited longer intensive care unit (ICU) (9 vs. 4 days, *p* < 0.001) and overall hospital stays (24 vs. 11 days, *p* < 0.001), despite similar post-operative hemodynamics achieved compared to non-sarcopenic patients. Conclusions: CTEPH patients with sarcopenia have worse baseline functional class and hemodynamics. For those with sarcopenia requiring surgery, there is longer ICU and total hospitalization stays, but they achieve significant functional improvements and hemodynamics comparable to that of non-sarcopenic patients. Hence, the risk of longer perioperative hospitalization days is justified by the longer-term benefit of hemodynamic improvement. The use of PMI as part of routine pre-operative assessments could improve clinical decision-making in CTEPH patients undergoing surgical or medical intervention.

## 1. Introduction

Sarcopenia is defined as the progressive loss of muscle strength, mass, and function, [1], leading to a decline in functional capacity, and it can be exacerbated by aging and chronic comorbidities [2,3]. It is often associated with a faster progression of cardiovascular disease, morbidity, and mortality [2,4,5,6,7,8]. Sarcopenia has not been investigated in patients with chronic thromboembolic pulmonary hypertension (CTEPH). CTEPH is a rare complication of pulmonary embolism characterized by the presence of chronic residual thrombi and fibrous stenosis of pulmonary arteries [9]. The obstruction of pulmonary arteries (PA) by organized clot formation leads to pathophysiological changes in the pulmonary vascular bed, increasing pulmonary vascular resistance (PVR) and frequently progressing to right-sided heart failure [10,11]. These sequelae manifest symptomatically as dyspnea, fatigue, exercise intolerance, and the presence of clinical heart failure [12]. Skeletal muscle wasting is increasingly being appreciated as contributing to the exercise intolerance observed in CTEPH [13]. Despite the estimated prevalence of sarcopenia being 31% in cardiovascular disease and 27% in respiratory disease populations, there is no literature regarding the value of the psoas muscle index (PMI) as a prognostic factor in CTEPH [14]. Therefore, we aimed to evaluate the incidence of sarcopenia in CTEPH and compare outcomes between sarcopenic and non-sarcopenic cohorts undergoing surgical pulmonary thromboendarterectomy (PTE) and balloon pulmonary angioplasty (BPA).

## 2. Methods

This study was a single-center retrospective analysis. We identified patients with CTEPH from 2019 to 2023 at Temple University Hospital. We included patients with CTEPH with an available pre-intervention CT abdomen–pelvis (CTAP). We defined ‘intervention’ as individuals requiring PTE or BPA. The psoas muscle area was measured at the L3 level of the CTAP scan, and PMI was then calculated using the formula: (left psoas area + right psoas area)/height^2^.

Exclusion criteria included the following: (1) patients less than 18 years of age; (2) patients without 3 months of follow-up information after intervention; and (3) patients without available pre-intervention CTAP. Our study was approved by the Internal Review Board (IRB) (IRB protocol number 27520) and the Research Review Committee (RRC).

Study data were collected and managed using REDCap (Research Electronic Data Capture) electronic data capture tools hosted at Temple University Hospital. We collected information regarding patient demographics, comorbidities, social history, date of diagnosis, WHO/NYHA functional class, baseline and post-intervention 6 min walk distance (6MWD), baseline and post-intervention hemodynamics, brain natriuretic peptide (BNP), and intervention treatment modality. The principal outcome measures gathered included change in baseline to post-intervention 6MWD (∆6MWD), BNP, and mortality. We also evaluated overall inpatient length of stay (LOS) and intensive care unit (ICU) LOS in patients undergoing PTE.

Every case was reviewed by an expert multidisciplinary CTEPH group that carefully reviewed patient history, comorbidities, imaging, and hemodynamics. The patient selection for PTE or BPA was performed in a systematic fashion.

### 2.1. Determination of Sarcopenia by Imaging

Pre-intervention CTAP scans were accessed using eRAD PACS Viewer version 7.2. PMI was obtained by measuring the cross-sectional area of bilateral psoas muscles at the level of L3. PMI was calculated as the psoas muscle area divided by the squared patient height in units of cm^2^/m^2^. Presently, there is no consensus regarding optimal PMI cutoff values for sarcopenia, which vary in different populations [15]. In this study, sarcopenia was defined as the lowest sex-specific quartile PMI at pre-intervention CTAP scan, in accordance with previous studies [16,17,18]. Patients in the 1st quartile of PMI for their sex (<5.27 cm^2^/m^2^ for male, <3.69 cm^2^/m^2^ for female) were classified as sarcopenic patients (SPs).

### 2.2. Statistical Analysis

The characteristics of the two cohorts (non-sarcopenic and sarcopenic) were compared using T-tests and the chi-squared test. A pre-specified 2-sided alpha of 0.05 and 95% Confidence Intervals (CIs) were used to determine statistical significance. Non-parametric tests, such as the Mann–Whitney U test, were used to compare variables that were not normally distributed. All statistical analyses were performed using IBM SPSS Statistics Version 29.0.0.0 (IBM Corp. Released 2022. IBM SPSS Statistics for Windows, Version 29.0. Armonk, NY, USA: IBM Corp.).

## 3. Results

### 3.1. Cohort Demographics

**Figure 1:**
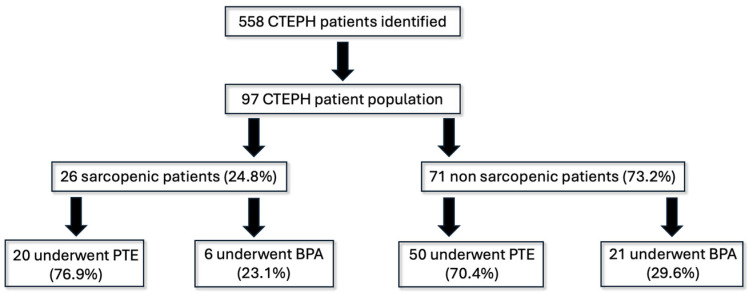
Cohort demographics.

We identified 558 cases of CTEPH from 2019 to 2023, of which we included 97 patients with pre-intervention CTAP. Sarcopenia was present in 26 (24.8%) of the patients with CTEPH. The cohort was divided into 28 patients in the SP cohort and 85 patients in the non-sarcopenic cohort (non-SP) (Figure 1).

Baseline characteristics and comorbidities are displayed in Table 1. There were no statistically significant differences between the SP and non-SP cohorts regarding age (64 ± 14 vs. 60 ± 15.8; *p* = 0.5), male sex (42% vs. 44%; *p* = 0.9); there was a trend toward lower Body Mass Index (BMI) in the SP group versus non-SP group (28.9 ± 9.4 vs. 32.7 ± 8.6 mg/kg^2^; *p* = 0.057). There were no significant differences between the SP and non-SP cohorts regarding comorbidities, including atrial fibrillation (18.3 vs. 19.2%, *p* = 0.9), history of cancer (3.8 vs. 12.7%, *p* = 0.3), coagulopathy (11.5 vs. 16.9%, *p* = 0.5), diabetes mellitus (15.4 vs. 18.3%, *p* = 0.7), family history of thromboembolic disease (3.8 vs. 4.2%, *p* = 0.9), hemoglobinopathy (3.8 vs. 1.4%, *p* = 0.45), history of acute pulmonary embolism (80.8 vs. 80.3%, *p* = 0.9), lower-extremity deep vein thrombosis (LE DVT) (53.8 vs. 69.2%, *p* = 0.6), upper extremity DVT (3.8 vs. 1.4%, *p* = 0.4), intravenous device (11.5 vs. 7%, *p* = 0.4), May–Thurner syndrome (7.7% vs. 8.5%, *p* = 0.9), history of splenectomy (3.8% vs. 1.4%, *p* = 0.4), sleep apnea (26.9 vs. 15.5%, *p* = 0.2), thyroid disease (19.2 vs. 11.3%, *p* = 0.3), or tobacco use (26.9 vs. 43.7%; *p* = 0.1).

**Figure 2:**
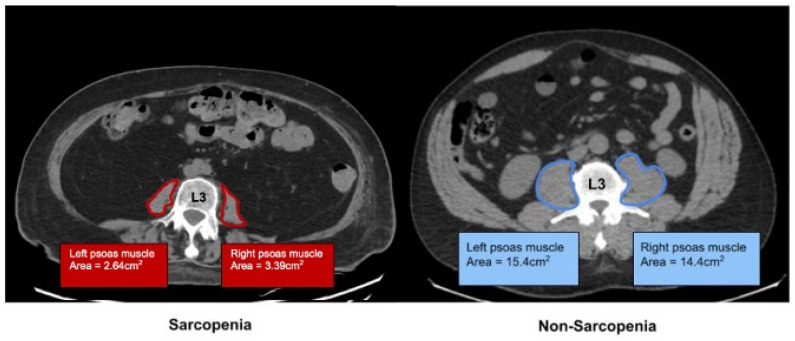
Psoas muscle index (PMI) a cross-sectional measurement of the bilateral psoas muscles at the level of the L3.

The PMI was lower in the SP cohort than in the non-SP cohort (3.24 vs. 6.11, *p* < 0.001). (Figure 2) The SP cohort was more likely to present with a worse WHO/NYHA functional class (FC) compared to the non-SP cohort (FC I, 0 vs. 12.7%; FC II, 11.5 vs. 29.6%; FC III, 42.3 vs. 43.7%; FC IV, 42.6% vs. 14.1%, *p* = 0.002). Despite not reaching statistical significance, there was a trend toward a lower baseline 6MWD in the SP cohort (289 vs. 343 m, *p* = 0.19).

The baseline median BNP was 138 picograms/mL (IQR 54–425). In the SP group, the baseline median BNP was significantly higher: 190 pg/mL (IQR 104–1008) vs. 131 pg/mL (IQR (41–353) (*p* = 0.03).

Baseline hemodynamics of the patients are present in Table 2. There were significant differences in the SP and non-SP groups evidenced by higher systolic PA pressures (83.5 vs. 68.1 mmHg, *p* = 0.003), diastolic PA pressures (28.4 vs. 23.9 mmHg, *p* = 0.02), mean PA pressure (48.5 vs. 39.8 mmHg), *p* = 0.002), PVR (9.8 vs. 6.9 WU, *p* = 0.009), and TPR (13.3 vs. 9.7 WU, *p* = 0.003). There were no statistical differences in the hemodynamics of patients undergoing PTE or BPA (Supplemental Table S1).

To assess whether sarcopenia was independently associated with post-operative outcomes beyond baseline biomarker severity, we conducted multivariable linear regression analyses for both ICU and overall hospital length of stay. Each model included sarcopenia status, baseline BNP, and age as covariates. Sarcopenia remained significantly associated with both longer ICU LOS (B = 11.53 days; 95% CI: 1.88–21.19; *p* = 0.021) and longer overall hospital LOS (B = 16.27 days; 95% CI: 6.34–26.19; *p* = 0.002). In contrast, BNP and age were not significant predictors in either model. These findings are summarized in Supplemental Table S2.

Of the entire cohort, 70 (72.2%) underwent PTE, and 27 (27.8%) underwent BPA. PH medical therapy was used in 42 (43.3%) of the entire cohort, and the SP cohort was more likely to be on pulmonary vasodilators at baseline (62 vs. 37%, *p* = 0.028), particularly with Sildenafil (28.0% vs. 6.9%, *p* = 0.006). The use of Riociguat and Macitentan did not significantly differ between groups.

Taken together, the presence of higher mPAP, PVR, greater use of PH medical therapy, and higher BNP values speak to a generally more advanced level of pulmonary hypertension and right heart dysfunction in the SP group versus the non-SP group.

### 3.2. Post-Intervention

**Figure 3:**
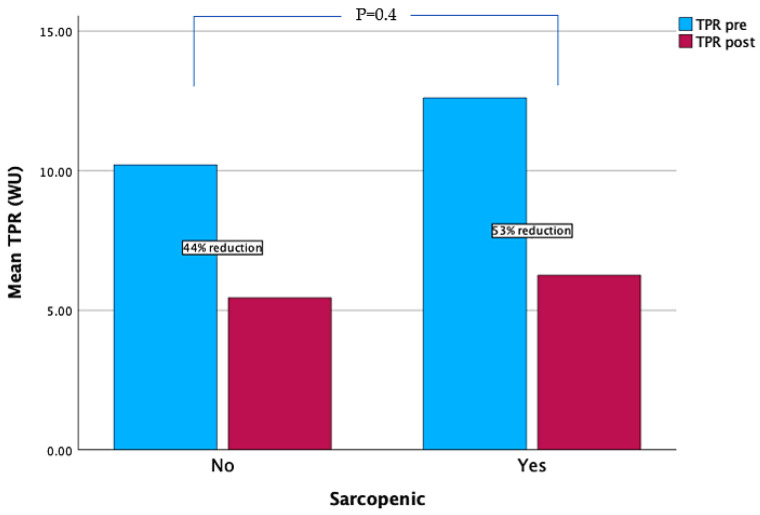
Changes in total pulmonary resistance.

Early post-intervention hemodynamics are summarized in Table 3. In the patients undergoing PTE the hemodynamics were obtained within 48–72 h post-surgery, and in the patients undergoing BPA, the hemodynamics were obtained 3 months after the last BPA session. After interventions, there was no longer a distinction between the SP and non-SP cohorts regarding mPA (30 vs. 27 mmHg; *p* = 0.84) and TPR (6.3 vs. 5.5 WU, *p* = 0.25). After intervention, there was a 33% decrease in mean PA and a 47% decrease in TPR. The SP cohort had a 53% decrease in TPR versus a 44% reduction in the non-SP cohort (*p* = 0.4) (Figure 3). There were no significant differences in the post-intervention hemodynamics between the group that underwent BPA or PTE.

Post-intervention biomarkers and 6MWD were obtained at the 3-month follow-up (Table 4). Post-intervention, there was an improvement of 94 pg/mL in BNP in the SP group and 63 pg/mL in the non-SP group. There was no mortality (0%) in either the sarcopenic or non-sarcopenic cohorts. The SP group had a 76 m increase in the 6MWD versus 18 m increase in the non-SP group.

### 3.3. Clinical Course, Biomarkers, 6MWD, and Post-PTE Specific Outcomes

**Figure 4:**
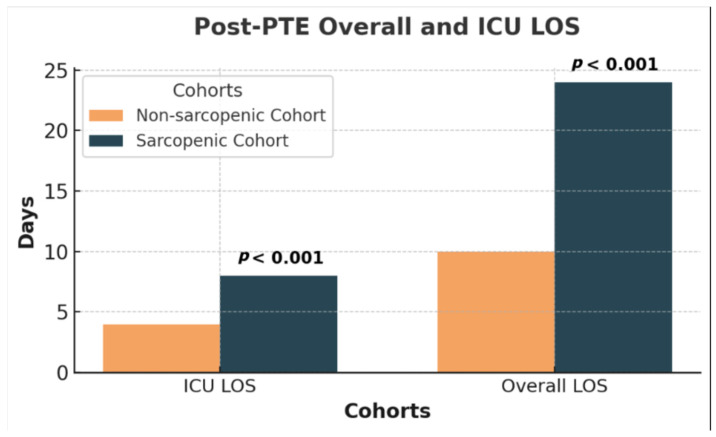
Post-PTE overall and ICU LOS.

The SP cohort was observed to have significantly longer average ICU LOS following PTE than the non-SP cohort (9 vs. 4 days; *p* < 0.001) as well as longer average overall inpatient LOS (24 vs. 11 days; *p* < 0.001). (Figure 4) Odds Ratios (ORs) were calculated to analyze the risk of exposure to age > 70, sarcopenic status, and baseline 6MWD < 300 m on overall LOS > 10 days following PTE. This analysis revealed that the OR for sarcopenia was higher than age > 70 or baseline 6MWD < 300 (OR 7.74, CI 1.6–36.9; OR 2.42, CI 0.7–8.3; OR 1.62, CI 0.51–5.1)

**Figure 5:**
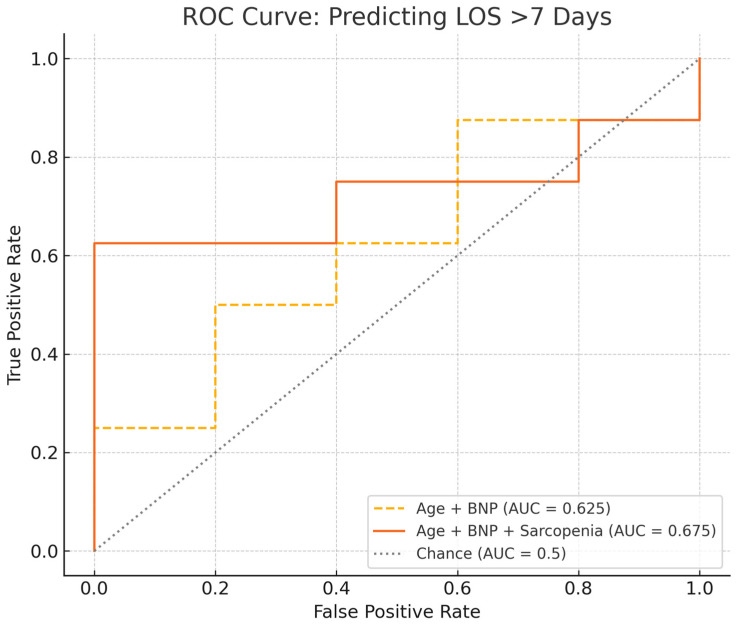
ROC curve comparing models with and without sarcopenia for predicting prolonged hospitalization.

To further evaluate the added prognostic value of sarcopenia, we constructed logistic regression models using LOS >7 days as a binary outcome. The baseline model including age and BNP yielded an AUC of 0.625, while the addition of sarcopenia improved the AUC to 0.675. These findings are displayed in Figure 5.

## 4. Discussion

This study is the first to examine PMI as a prognostic factor in CTEPH patients. While prior studies have defined sarcopenia via hand grip strength and other non-radiologic markers, PMI offers the advantage of being an objective, quantitative, and easily derived measure in studies. Sarcopenic patients had more advanced diseases, as indicated by greater proportion of NYHA Class IV functional status, as well as more severe preoperative hemodynamic parameters, including higher mean PA pressures and TPR. In keeping, the SPs were also far more likely to be treated with PH medical therapy prior to intervention. Notably, following PTE or BPA, there was no longer a distinction between mean PA pressures and TPR, indicating that sarcopenic patients have improvements in overall hemodynamics post-procedure. Moreover, these subjects experienced a greater relative improvement in hemodynamics post-intervention, as well a higher improvement in the meters achieved in their 6MWD.

In terms of post-operative PTE outcomes, sarcopenia emerged as a strong predictor of prolonged ICU and overall LOS, aligning with prior literature demonstrating that PMI-derived sarcopenia is associated with increased morbidity in the perioperative period due to frailty [17]. This was further supported by ROC analysis, in which the addition of sarcopenia to a model including age and BNP modestly improved discrimination for predicting prolonged hospitalization (AUC 0.675 vs. 0.625). This finding is consistent across multiple surgical specialties, including cardiothoracic and thoracic surgery, where sarcopenia has been shown to increase resource utilization [18,19]. It seems to be valuable to assess perioperative morbidity and administrative measurements. Importantly, our analysis found that sarcopenia posed a greater risk for prolonged LOS after PTE than other established pre-operative risk factors, such as age greater than 70 years or baseline 6MWD less than 300 m. This highlights the predictive value of PMI in the CTEPH population for longer hospital course, offering a more precise prediction tool than traditional metrics like 6MWD which are volitional [20,21].

Post-intervention functional improvement, as measured by 6MWD, was notable in both sarcopenic and non-sarcopenic patients. The SP cohort demonstrated significant functional gains post-intervention, with a far greater improvement in 6MWD post-intervention than in the non-SP group. This suggests that in the setting of appropriate patient selection, sarcopenic patients may derive even greater substantial benefit from PTE or BPA despite their poorer baseline functional status.

The majority of sarcopenic patients in this cohort were considered suitable for PTE, as their musculoskeletal deconditioning was likely attributable to the hemodynamic severity of their condition rather than other significant comorbidities. The sarcopenic patients had worse baseline hemodynamics and BNP, both suggesting worse right heart dysfunction. After interventions, SPs may be poised to a greater hemodynamic improvement, and this may explain the nearly half reduction in BNP and the higher percentage gain in the 6MWD.

Long-term improvement in exercise capacity following PTE can be expected, with previous studies indicating sustained gains in 6MWD for up to five years [4,22]. The degree to which sarcopenic patients can recover skeletal muscle mass post-intervention remains an area of interest. Prospective studies are warranted to explore how skeletal muscle mass evolves following PTE and BPA, with particular attention to the potential for long-term functional improvement. Future investigations should also consider tracking PMI measurements pre- and post-intervention to better understand the relationship between CTEPH severity, sarcopenia, and exercise capacity over time.

Screening and testing for sarcopenia is important in patients with chronic disease states, as it may provide an opportunity for interventions to reverse or delay progression. There are various methods of screening for sarcopenia, such as the SARC-F (Strength, Assistance walking, Rise from a Chair, Climb Stairs, and Falls) questionnaire, Ishii et al.’s screening tool, the MSRA (Mini-Sarcopenia Risk Assessment) questionnaire, SarSA-Mod (Sarcopenia Scoring Assessment Models), chair rise, and hand grip strength [23]. There is no current consensus on the best tools for screening sarcopenia in clinical practice. To establish the diagnosis, one requires confirmatory testing which includes laboratory tests, physical performance tests (e.g., gait speed), imaging, and body composition analysis (i.e., CT scans or magnetic resonance imaging and dual-energy x-ray absorptiometry) [24,25]. The gold standard is the utilization of instruments to quantify muscle mass and provide precise and direct visualization of body compartments [1]. Historically, this entailed the evaluation of the cross-sectional muscle area (CSMA) of the psoas major, erector spinae, transverse abdominis, quadratus lumborum, obliques, and rectus abdominus at the third lumbar level (L3) [26,27].

Recently, single-muscle measurements for the diagnosis of sarcopenia have emerged as an alternative to total CSMA. The psoas major muscle is most frequently utilized given its function as the main hip flexor responsible for postural support of the spine and hip and its ease of identification on CT imaging [28]. PMI, a cross-sectional measurement of the bilateral psoas muscles at the level of L3, is correlated with lean body skeletal muscle mass and has been shown to predict outcomes in a range of cardiac and oncological pathologies [29,30,31], as well as in patients undergoing various surgical and minimally invasive procedures [32]. A prior abstract by Balki et al. demonstrated the relevance of sarcopenia in patients undergoing pulmonary endarterectomy for CTEPH; however, their study did not incorporate quantitative imaging-based muscle assessment or examine functional recovery [33]. We were able to demonstrate the ease of reproducibility of PMI in our cohort, supporting its broader clinical applicability.

We had several limitations in this study. Firstly, the retrospective design of the study introduces inherent biases and limitations in data collection. Furthermore, although patients with known active malignancies were excluded, subclinical or undiagnosed cancer may have been present and could confound the observed associations. Prospective studies with larger sample sizes and standardized protocols must validate these findings. In addition, CTAP is not a standard test in the evaluation of patients with CTEPH, this affected the sample size and statistical power of our study. In fact, the clinical indications that warranted CTAP in our CTEPH cohort may have introduced bias. The measurements of PMI were performed free hand, without an automated software, which can lead to inter-operator variability. To overcome this, we carried out an interobserver analysis demonstrating good agreement using Fleiss’ Kappa statistics. Lastly, all BPA patients only had a 24 h stay post-procedure as per standard of care; therefore, LOS is a metric for PTE patients.

Future research should aim to incorporate additional clinical measurements of frailty to provide a complete assessment of sarcopenia. Prospective studies should evaluate the comparative prognostic utility of PMI and biochemical nutritional markers such as albumin and prealbumin in CTEPH patients undergoing intervention. We did not perform IDI or NRI analyses due to the small sample size and limited number of binary outcome events, which would limit the interpretability and reliability of such reclassification metrics. Future studies with larger cohorts should explore the incremental predictive utility of sarcopenia using these advanced comparative models. Future studies should also assess whether PMI-derived sarcopenia predicts long-term mortality, functional decline, and clinical deterioration beyond the perioperative period. Importantly, the study population was limited to patients referred to a specific hospital’s Pulmonary Hypertension, Right Heart Failure, and CTEPH Program. The findings may not be representative of the broader CTEPH population as the expert multidisciplinary CTEPH team was very careful with patient selection. Conducting multi-center studies involving diverse patient populations would enhance the generalizability of the results.

## 5. Conclusions

Sarcopenia was associated with worse baseline hemodynamics and functional class, as well as longer ICU and hospital length of stay. PMI-derived sarcopenia may serve as a useful adjunct for identifying patients at risk for more severe preoperative disease and longer perioperative recovery. Despite these differences, sarcopenic and non-sarcopenic patients achieved comparable improvements in post-intervention functional capacity and hemodynamics. These findings support the use of PMI in preoperative risk stratification, while also reinforcing the value of intervention even in patients with sarcopenia. A proposed clinical workflow is presented to illustrate how PMI can be integrated into existing multidisciplinary evaluation pathways.

## Figures and Tables

**Table 1:** Cohort demographics, characteristic, and 6MWD.

	All (N = 97) Mean (±SD) or N (%)	Sarcopenic (N = 26) Mean (±SD) or N (%)	Non-Sarcopenic (N = 71) Mean (±SD) or N (%)	OR 95% CI	*p* Value
Age (years)	61.3 (±14.9)	63.5 (±13.9)	60 (±15.8)		0.52
BMI (mg/kg^2^)	31.7 (±29.5)	28.9 (±9.4)	32.7 (±8.6)		0.057
Sex (male)	42 (43.3%)	11 (42%)	31 (43.7%)	0.95 (0.38–2.34)	0.9
Race/Ethnicity:					
White	51 (52.6%)	18 (69.2%)	33 (46.5%)		
Black	38 (39.2%)	7 (26.9%)	31 (43.7%)		
Hispanic/Latinx	7 (7.2%)	1 (3.8%)	6 (8.5%)		
Asian	1 (1%)	0 (0%)	1 (1.4%)		0.2
Baseline WHO/NYHA FC					
I	9 (9.3%)	0 (0%)	9 (12.7%)		
II	24 (24.7%)	3 (11.5%)	21 (29.6%)		
III	42 (43.3%)	11 (42.3%)	31 (43.7%)		
IV	22 (22.7%)	12 (42.6%)	10 (14.1%)		0.002
Comorbidities					
Use of PH Medications	42 (43.3%)	16 (61.5%)	26 (36.6%)	2.7 (1.09–6.99)	0.028
- Sildenafil	12 (12.4%)	7 (28.0%)	5 (6.9%)		0.006
- Riociguat	21 (21.6%)	8 (32.0%)	13 (18.0%)		0.145
- Macitentan	6 (6.2%)	2 (8.0%)	4 (5.6%)		0.662
Atrial Fibrillation	18 (18.6%)	5 (19.2%)	13 (18.3%)	1.06 (0.34- 3.34)	0.91
Cancer History	10 (10.3%)	1 (3.8%)	9 (12.7%)	0.28 (0.03–2.29)	0.21
Coagulopathy	15 (15.5%)	3 (11.5%)	12 (16.9%)	0.64 (0.17–2.48)	0.51
DM	17 (17.5%)	4 (15.4%)	13 (18.3%)	0.81 (0.24–2.76)	0.74
Family History of Thromboembolic disease	4 (4.1%)	1 (3.8%)	3 (4.2%)	0.91 (0.09–9.13)	0.93
Hemoglobinopathy	2 (2.1%)	1 (3.8%)	1 (1.4%)	2.8 (0.17–46.46)	0.45
History of Acute PE	78 (80.4%)	21 (80.8%)	57 (80.3%)	1.03 (0.33–3.22)	0.96
History of LE DVT	56 (57.7%)	14 (53.8%)	42 (59.2%)	0.81 (0.34–1.99)	0.64
History of UE DVT	2 (2.1%)	1 (3.8%)	1 (1.4%)	2.8 (0.17–46.47)	0.45
IV Device	8 (8.2%)	3 (11.5%)	5 (7%)	1.72 (0.38–7.78)	0.47
MTS	8 (8.2%)	2 (7.7%)	6 (8.5%)	0.90 (0.17–4.78)	0.94
Splenectomy	2 (2.1%)	1 (3.8%)	1 (1.4%)	2.8 (0.17–46.46)	0.45
Sleep apnea	18 (18.6%)	7 (26.9%)	11 (15.5%)	2.01 (0.68–5.91)	0.20
Thyroid disease	13 (13.4%)	5 (11.3%)	8 (19.2%)	1.88 (0.55–6.36)	0.31
Tobacco use	38 (39.2%)	7 (26.9%)	31 (43.7%)	0.47 (0.18–1.27)	0.14
Psoas Muscle Index (PMI)	5.22 (±2.87)	3.24 (±0.75)	6.11 (±1.18)		<0.001
Baseline 6MWD (meters)	332.3 (±134.6)	289.3 (±102.2)	343.8 (±140.5)	0.4 (−0.16–0.98)	0.19

Abbreviations: BMI—Body Mass Index; CI—Confidence Interval; DM—diabetes mellitus; DVT—deep vein thrombosis; IV—Intravenous; LE—lower extremity; MTS—May–Thurner syndrome; NYHA—New York Heart Association; OR—Odds Ratio; PE—pulmonary embolism; PMI—psoas muscle index; PH—pulmonary hypertension; UE—upper extremity; WHO—World Health Organization; 6MWD—6 min walk distance.

**Table 2:** Baseline hemodynamics of the cohort.

Hemodynamics	All N = 97 Mean (±SD)	Sarcopenic N = 26	Non-Sarcopenic N = 71	*p* Value
Heart Rate (bpm)	78 (±13.1)	78.4 (±10.5)	78.3 (±14.2)	0.97
Right atrial pressure (mmHg)	9.9 (±5.2)	9.8 (±5)	9.9 (±5.3)	0.93
Systolic PA Pressure (mmHg)	72.3 (±23.2)	83.5 (±19.6)	68.1 (±23.2)	0.003
Diastolic PA Pressure (mmHg)	25.1 (±8.9)	28.4 (±7.6)	23.9 (±9.1)	0.02
Mean PA (mmHg)	42.2 (±12.5)	48.5 (±11.3)	39.8 (±12.2)	0.002
PCWP (mmHg)	12.1 (±4.8)	11.6 (±4.4)	12.2 (±4.9)	0.58
CO (L/min)	4.41 (±1.25)	4.08 (±1.25)	4.53 (±1.23)	0.11
CI (L/min/m^2^)	2.18 (±0.57)	2.04 (±0.57)	2.24 (±0.56)	0.12
PVR (WU)	7.76 (±4.61)	9.79 (±4.45)	6.98 (±4.46)	0.009
TPR (WU)	10.67 (±5.26)	13.26 (±5.53)	9.67 (±4.83)	0.003

Abbreviations: BPM—beat per minute; CI—cardiac index; CO—cardiac output; mmHg—millimeters of mercury; PA—pulmonary artery; PCWP—pulmonary capillary wedge pressure; PVR—pulmonary vascular resistance; SD—standard deviation; TPR—total pulmonary resistance; WU—Wood Units.

**Table 3:** Early post-intervention hemodynamics of patients receiving PTE or BPA.

Hemodynamics	All N = 97 (±SD)	Sarcopenic N = 26	Non-Sarcopenic N = 71	*p* Value
Systolic PA Pressure (mmHg)	43.1 (±15.5)	49.1 (±18.1)	44.4 (±15.3)	0.99
Diastolic PA Pressure (mmHg)	17.7 (±6.6)	20.1 (±6.1)	18.7 (±6.3)	0.90
Mean PA pressure (mmHg)	28.6 (±8.9)	30.4 (±9.7)	27.2 (±8.6)	0.84
CO (L/min)	5.56 (±1.29)	5.5 (±1.95)	5.4 (±1.25)	0.09
CI (L/min/m^2^)	2.67 (±0.52)	2.56 (±0.54)	2.7 (±0.53)	0.06
TPR (WU)	5.61 (±2.25)	6.25 (±2.77)	5.45 (±2.13)	0.27

Abbreviations: mmHg—millimeters of mercury; PA—pulmonary artery; SD—standard deviation; TPR—total pulmonary resistance; WU—Wood Units.

**Table 4:** Biomarkers, 6 min walk distance, and ICU LOS Post-PTE.

Variables	All N = 97	Sarcopenic N = 26	Non-Sarcopenic N = 71	*p* Value
6MWD (meters) Pre-Intervention	Mean (SD) 332.3 (±134.6)	Mean (SD) 289.3 (±102.2)	Mean (SD) 343.8 (±140.5)	0.22
Post-Intervention	362.2 (±118.3)	365.2 (±140.8)	361.4 (±113)	0.69
Brain natriuretic peptide (pg/mL) Pre-Intervention	Median (IQR) 138.5 (IQR 54–428)	Median (IQR) 190 (IQR 104–1008)	Median (IQR) 129 (IQR 37–353)	0.03
Post Intervention	71 (IQR 36–144)	96 (IQR 41–242)	66 (IQR 35–117)	0.22
Post-PTE ICU LOS (days)	4 (p25 = 3, p75 = 10)	9 (p25 = 5, p75 = 34)	4 (p25 = 2, p75 = 7)	<0.001
Post-PTE Overall LOS (days)	14 (p25 = 7, p75 = 24)	24 (p25 = 12, p75 = 41)	11 (p25 = 7, p75 = 17)	<0.001
Mortality	0	0	0	

Abbreviations: 6MWD—6-min walk distance; ICU LOS—Intensive Care Unit Length of Stay; IQR—Interquartile Range; LOS—length of stay; P25—25th Percentile; P75—75th Percentile; PMI—psoas muscle index; PTE—pulmonary thromboendarterectomy.

## Data Availability

The datasets generated and/or analyzed during the current study are not publicly available due to patient confidentiality but are available from the corresponding author on reasonable request.

## Supplementary Materials

The following supporting information can be downloaded at: https://www.mdpi.com/article/10.3390/jcdd12050162/s1, Figure S1: Clinical Decision Algorithm: PMI-Based Sarcopenia Assessment in CTEPH; Table S1: Baseline hemodynamics for the entire cohort divided by PTE and BPA; Table S2: Multivariable Regression Results for ICU and Total Hospital Length of Stay.

## Abbreviations

The following abbreviations are used in this manuscript:

Abbreviation	Definition
BMI	Body Mass Index
BPA	Balloon Pulmonary Angioplasty
CI	Confidence Interval
CO	Cardiac Output
CTAP	Computed Tomography Abdomen/Pelvis
CTEPH	Chronic Thromboembolic Pulmonary Hypertension
DOAC	Direct Oral Anticoagulant
DVT	Deep Vein Thrombosis
FC	Functional Class
FFP	Fractional Pulse Pressure
HGS	Hand Grip Strength
ICU	Intensive Care Unit
IRB	Institutional Review Board
LE DVT	Lower-Extremity Deep Vein Thrombosis
LOS	Length of Stay
mPA	Mean Pulmonary Artery Pressure
MTS	May–Thurner Syndrome
NYHA	New York Heart Association
OR	Odds Ratio
PCWP	Pulmonary Capillary Wedge Pressure
PE	Pulmonary Embolism
PH	Pulmonary Hypertension
PMI	Psoas Muscle Index
PTE	Pulmonary Thromboendarterectomy
PVR	Pulmonary Vascular Resistance
REDCap	Research Electronic Data Capture
ROS	Reactive Oxygen Species
SP	Sarcopenic Patient
TPR	Total Pulmonary Resistance
UE DVT	Upper-Extremity Deep Vein Thrombosis
WHO	World Health Organization
