# Supplementary material for: Sarcopenia in Patients with Chronic Thromboembolic Pulmonary Hypertension
Source: J Cardiovasc Dev Dis. 2025 Apr 22;12(5):162. doi: 10.3390/jcdd12050162 (PMC12112501; doi:10.3390/jcdd12050162)

Supplemental Table S1. Baseline hemodynamics for the entire cohort divided by PTE and BPA

	All<br>N= 97<br>Mean (± SD)	PTE<br>N= 70	BPA<br>N= 27	p value
Heart Rate (bpm)	78 (± 13.1)	80 (±12.2)	75 (±14.5)	0.17
Right atrial pressure (mmHg)	9.9 (± 5.2)	10.3 (±5.2)	9.2 (±5.2)	0.38
Systolic PA Pressure (mmHg)	72.3 (± 23.2)	72.7 (±23.8)	71.4 (±22.05)	0.80
Diastolic PA Pressure (mmHg)	25.1 (± 8.9)	25.8 (±8.9)	23.2 (±8.7)	0.20
Mean PA pressure (mmHg)	42.2 (± 12.5)	42.7 (±12.6)	40.8 (±12.5)	0.53
PCWP (mmHg)	12.1 (± 4.8)	11.8 (±4.9)	12.6 (±4.4)	0.51
CO (L/min)	4.41 (± 1.25)	4.35 (±1.13)	4.56 (±1.53)	0.46
CI (L/min/m2)	2.18 (± 0.57)	2.14 (±0.52)	2.3 (±0.65)	0.21
PVR (WU)	7.76 (± 4.61)	8.03 (±4.8)	7.09 (±4.11)	0.38
TPR (WU)	10.67 (± 5.26)	10.89 (±5.49)	10.05 (±4.6)	0.49

Abbreviations: bpm – Beats per minute; mmHg – Millimeters of Mercury; CI- cardiac index; CO- cardiac output; PA – Pulmonary Artery; PCWP- pulmonary capillary wedge pressure; PVR – pulmonary vascular resistance; SD – Standard Deviation;
TPR – Total Pulmonary Resistance; WU – Wood Units

Supplemental Table S2. Multivariable Regression Results for ICU and Total Hospital Length of Stay

Dependent Variable	Independent Variable	Coefficient (B)	p-value	95% CI
ICU LOS	Sarcopenia	11.53	0.021	1.88 – 21.19
ICU LOS	BNP	-0.00	0.354	-0.01 – 0.01
ICU LOS	Age	0.01	0.96	-0.31 – 0.33
Total LOS	Sarcopenia	16.27	0.002	6.34 – 26.19
Total LOS	BNP	-0.01	0.083	-0.02 – 0.00
Total LOS	Age	0.08	0.623	-0.26 – 0.42

Abbreviations: Intensive Care Unite length of stay – ICU LOS; Total length of stay – total LOS; B-type natriuretic peptide- BNP

Supplemental Figure S1: Clinical Decision Algorithm: PMI-Based Sarcopenia Assessment in CTEPH

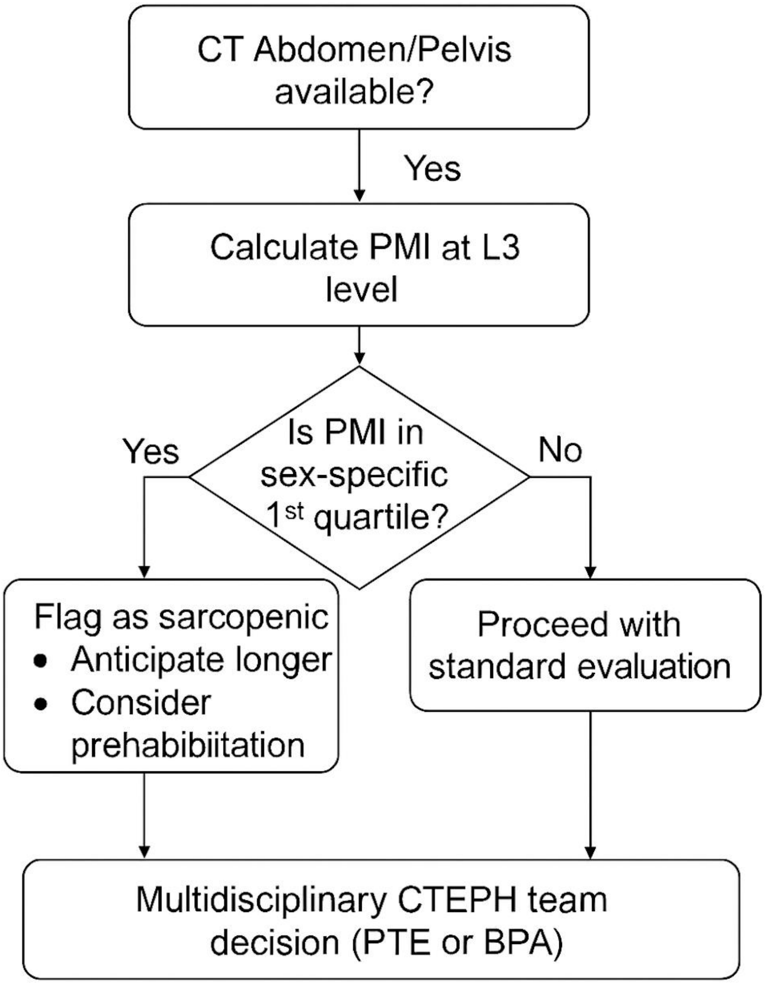

Supplement: Supplementary file 1 [file jcdd-12-00162-s001.zip › jcdd-3562915-supplementary.pdf]
